# Supplementary material for: Predicting visual function by interpreting a neuronal wiring diagram
Source: Nature. 2024 Oct 2;634(8032):113–23. doi: 10.1038/s41586-024-07953-5 (PMC11446822; doi:10.1038/s41586-024-07953-5)
Supplement: Supplementary file 6 — CRF and ERF predictions for individual TmY4 and TmY9 cells. Analogous to Supplementary Data 3, but for TmY target types. Shown are the top four monosynaptic pathways, the strongest pathway passing through each of the top ten intermediary types (ranking from Extended Data Fig. 7), and the trisynaptic pathway Tm1–TmY–Dm3–TmY (see the section entitled Prediction of spatial normalization). [file 41586_2024_7953_MOESM6_ESM.zip › DataS4/TmY9q/720575940636639374.pdf]

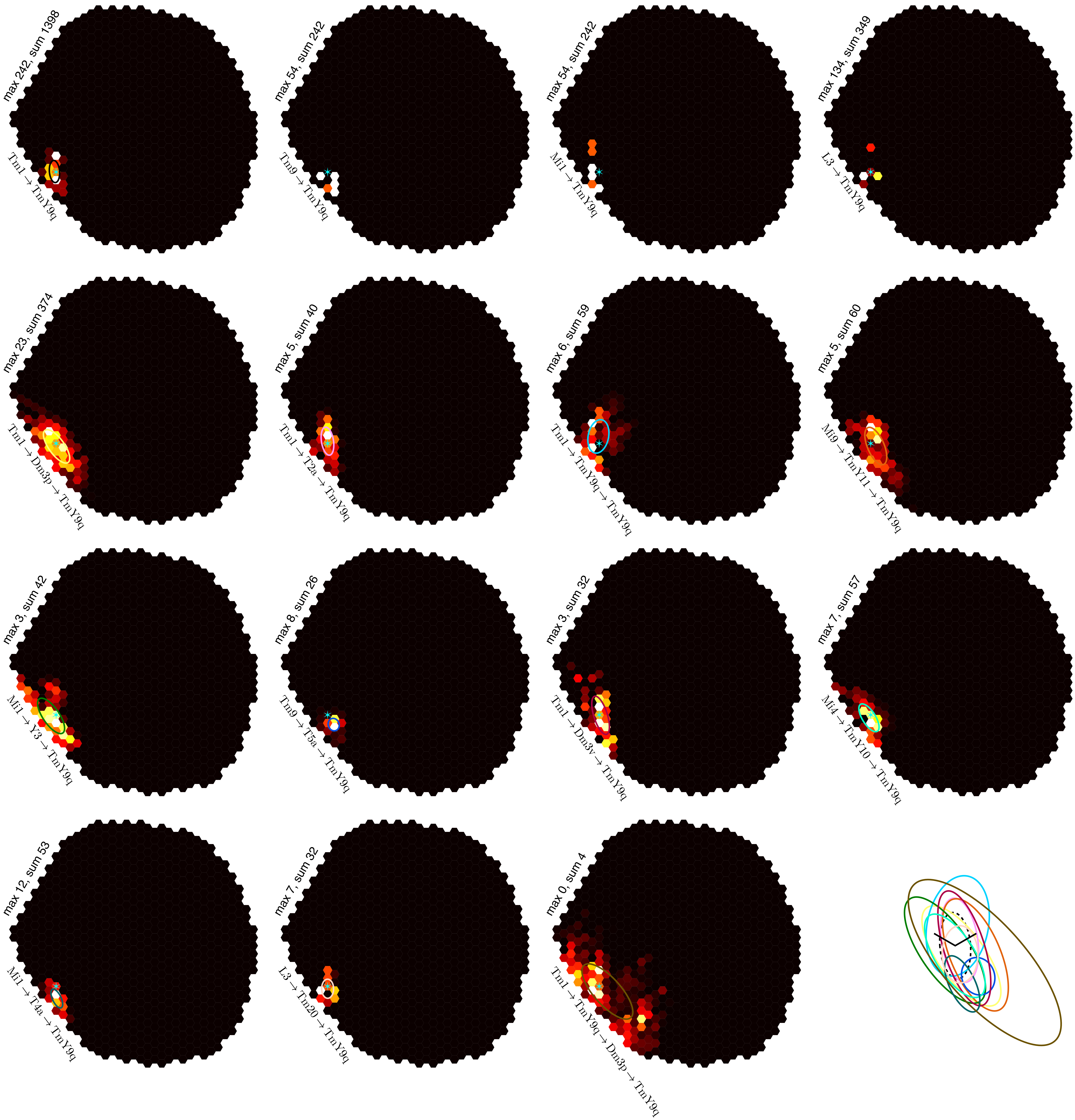
max 242, sum 1398
Tm1 → TmY9q
max 54, sum 242
Tm9 → TmY9q
max 54, sum 242
Mi1 → TmY9q
max 134, sum 349
L3 → TmY9q
max 23, sum 374
Tm1 → Dm3p → TmY9q
max 5, sum 40
Tm1 → T2a → TmY9q
max 6, sum 59
Tm1 → TmY9q → TmY9q
max 5, sum 60
Mi9 → TmY11 → TmY9q
max 3, sum 42
Mi1 → Y3 → TmY9q
max 8, sum 26
Tm9 → T5a → TmY9q
max 3, sum 32
Tm1 → Dm3v → TmY9q
max 7, sum 57
Mi4 → TmY10 → TmY9q
max 12, sum 53
Mi1 → T4a → TmY9q
max 7, sum 32
L3 → Tm20 → TmY9q
max 0, sum 4
Tm1 → TmY9q → Dm3p → TmY9q
